# Renaming COPD exacerbations: the UK respiratory nursing perspective

**DOI:** 10.1186/s12890-021-01662-9

**Published:** 2021-09-23

**Authors:** Christine Mwasuku, Joanne King, Richard E. K. Russell, Mona Bafadhel

**Affiliations:** 1 Respiratory Medicine Unit, Nuffield Department of Clinical Medicine, University of Oxford, Oxford, UK; 2 Respiratory NIHR Biomedical Research Centre, University of Oxford, Oxford, UK; 3 Windsor King Edward VII Hospital and Wexham Park Hospital Slough, Frimley NHS Foundation Trust, Windsor, UK

**Keywords:** COPD Crisis, Patients, Nurses, Exacerbation

## Abstract

**Background:**

Patients with COPD experience acute worsenings, termed ‘exacerbations’. While other terms to describe these events have been proposed there is no consensus on terminology which has led to multiple terms being used across the UK. Respiratory nurses are part of a multi-disciplinary team managing COPD patients, however, the nursing perspective on the term ‘exacerbation’ is unknown.

**Methods:**

An anonymised survey of 17 questions was sent to respiratory nurses through an email invitation link. The survey link was open for one month. The aim was to understand the nurse perspective on ‘exacerbation’. Alternative terms used in the UK were compared versus the term 'exacerbation’.

**Results:**

Responses were received from 113 nurses. The majority (88%) were female. There was no consensus on preference or meaning for the term ‘exacerbation’ between nurses. Less than 5% of nurses thought that patients with COPD would understand the term ‘exacerbation’. In ranked order, the nurses preferred the following terms: ‘flare-up’, ‘lung attack’, ‘crisis’, ‘exacerbation’ and ‘chest infection’. The term ‘crisis’, although new, was considered to be the term that most resonated with clinical practice.

**Conclusion:**

Respiratory nurses in the UK report that the term ‘exacerbation’ is not fit for purpose for patients, and alternatives should be sought.

**Supplementary Information:**

The online version contains supplementary material available at 10.1186/s12890-021-01662-9.

## Background

Chronic obstructive pulmonary disease (COPD) is an illness disrupted by acute and potential life-threatening respiratory episodes of worsened symptoms, termed ‘exacerbations’. These are managed most often by a multidisciplinary team, namely respiratory nurses and physiotherapists [1]. Patient understanding of the word ‘exacerbation’ is poor and the term is not liked [2]. Furthermore, patients who experience an ‘exacerbation’ equate the debilitating consequences in mental state, and quality of life, in similar degree as patients experiencing an acute coronary event [3]. However, coronary events have ‘heart attack’ as a universal and simple term for patients and healthcare professionals to understand. Heart attacks have positive patient outcomes compared to COPD exacerbations which is in part due to the terminology used [4]. It would therefore follow that improvement of health literacy would lead to an improvement of understanding ‘exacerbations’ and the terminology used to describe the episodes needs to convey the gravity of the situation for patients, carers and healthcare professionals alike.

A patient with COPD will encounter respiratory nurses at every stage of their disease course. Nurses are in a good position to affect change and improve patient outcomes. However, little is known about the respiratory nurse’s opinion on the term ‘exacerbation’. In attempts to redefine these acute episodes, several alternatives have been proposed by medical clinicians, including COPD ‘lung attack’[4], COPD ‘flare-up’ [5] and COPD ‘crisis’[6]. Currently there is no global consensus on which term to adopt. In this confidential survey of respiratory nurses, we explored beliefs on the term ‘exacerbation’ and alternative terms to use during acute events in COPD.

## Methods

### Respondents

An anonymised survey via the online platform Survey Monkey was performed. The survey was distributed to respiratory nurses in the United Kingdom (U.K) working with patients with COPD via the Association of Respiratory Nurses (ARNS) and Primary Care Respiratory Society (PCRS). The study link was live for one month beginning in March 2020 and resent once to ARNS members and twice to members of PCRS. All questions required an answer, with an estimated completion time of 10 min.

### Survey

Qualitative and quantitative questions were asked on use of the term ‘COPD exacerbation’ and how this compared to other terms (Additional file 1: Table S1). A total of 13 quantitative questions in the format of a Likert scale or ranking style were used. The remainder of the questions were qualitative, focusing on the responders understanding of the terms used in addition to the opinion on patient perspective. The survey was divided into: (1) responder demographical data including age, gender, years of experience, areas of work both geographically and speciality; (2) behavioural understanding of the responder definition of ‘exacerbations’; (3) level of understanding and experience of the responder to the term ‘exacerbation’; (4) introduction to new terms.

**Table 1:** Sub-themes of the theme ‘level of understanding of terms’

Sub-theme	Quote
‘Exacerbation’ is widely recognised in COPD	‘*Exacerbation is mentioned in the COPD care plan*’ and ‘pulmonary rehab mentions exacerbation’.
	‘*Patients understand but often don’t understand that they can just have a worsening of usual symptoms & therefore will associate with antibiotic use with exacerbation’*
Flare up is a familiar term	*‘Flare-up is easy to understand, it is a term some have heard used for Arthritis and Asthma’*
Patients may need to be taught what the term ‘exacerbation’ means	*‘Patients understand the term flare up much better’*
‘Chest infection’ is a misplaced term in describing ‘exacerbations’	*‘Exacerbation’- I like this as it leads nicely into explaining what the umbrella term means*.
	*‘I think chest infection is an inaccurate description of what is often going on’*
	*‘Avoid use of chest infection as implies need for antibiotics which are not always required’*
The use of ‘lung attack’ is widely promoted	*‘Flare-up is easy to understand, it is a term some have heard used for Arthritis and Asthma’*
	*‘Patients understand the term flare up much better’*
	*‘consistent language with the British lung foundation’*
‘Crisis’ is unfamiliar to respiratory nurses but it has potential to make an impact	*‘I like the term crisis- it isn’t all encompassing as in mild crisis?’*
	*Does work, you don’t really hear the term mild heart attack. It’s a heart attack or not’*

### Statistical analysis

Quantitative data were expressed as a whole number and percentages rounded off to the nearest whole number or to one decimal place. Means and standard deviation were calculated where appropriate. MAXQDA 2020 VERBI software was used to organise and analyse qualitative data [7] and thematic analysis was employed [8]. All qualitative responses were grouped into questions, that were similar using colour tags. They were then re-grouped into answers with similar responses. The answers were further categorised into uniting themes as determined by the authors. Ethical approval was not required for this survey in anonymised healthcare professionals.

## Results

### Respondents

There were 117 survey responses collected. Complete data from respiratory nurses was available from 113 responses. Females made up the majority of respondents (88%) and 70% were nurses working in either a primary or secondary healthcare setting. Clinical experience ranged from 1 to 32 years working with a mean (± SD) of 12 (± 8) years worked with patients who have COPD. The majority (81%) of respiratory nurses saw COPD patients as part of their routine clinical practice, greater than 50% of their time.

### ‘Exacerbation’ and other terms

**Fig. 1:**
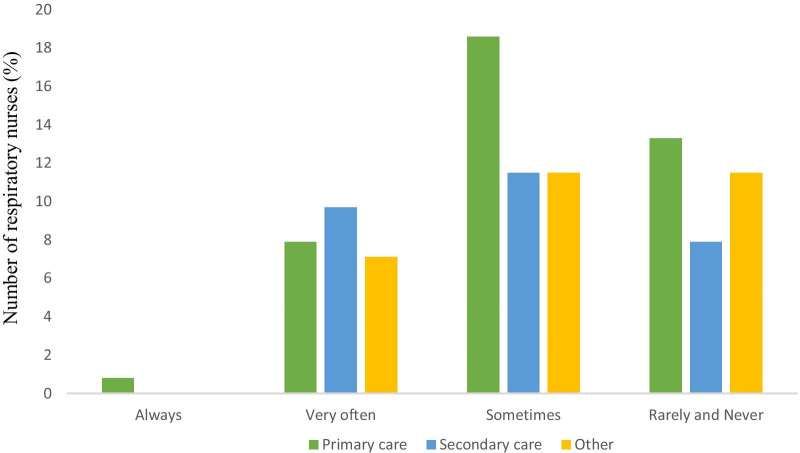
Bar graph illustrating the response to, how the term ‘exacerbation’ adequately describes the way a patient feels, sub-categorised by clinical setting. Respiratory nurses who do not work in primary or secondary care have been grouped as other

Respiratory nurses were asked to define an ‘exacerbation’. Overall, 77% recognised symptoms as a key part of an ‘exacerbation’ and 6% recognised that an ‘exacerbation’ was a limiting illness. In defining an ‘exacerbation’, commonly used words included: *increased* (as an adjective to describe symptoms such as cough, dyspnoea and breathlessness), *worsening* and *symptoms*. All terms used by respiratory nurses to describe these acute events that patients experience are presented as a word cloud in Additional file 1: Fig. S1. Furthermore, only 1 person reported the term ‘exacerbation’ adequately described the way a patient always feels; with the majority of nurses reporting the term ‘exacerbation’ was not expressive of how a patient feels at the time of deterioration (see Fig. 1). Only 4.4% of nurses thought patients with COPD fully understood the term ‘exacerbation’. All respiratory nurses reported they could not recall patients using the term ‘exacerbation’ spontaneously when describing their symptoms at time of acute illness.

**Fig. 2:**
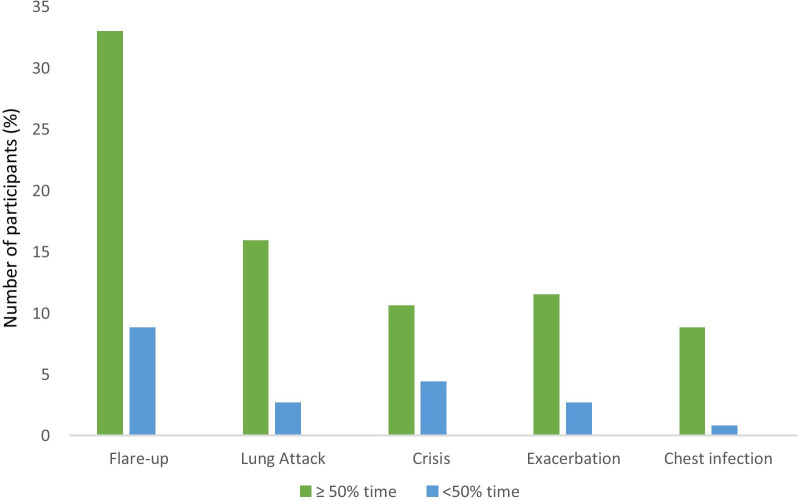
Bar graph in rank order of respiratory nurse responses to preferred use of words: ‘Flare-up, Lung attack, Exacerbation, Crisis, Chest infection’, sub-categorised by proportion of time spent seeing COPD patients (50% of their time including above and below)

**Fig. 3:**
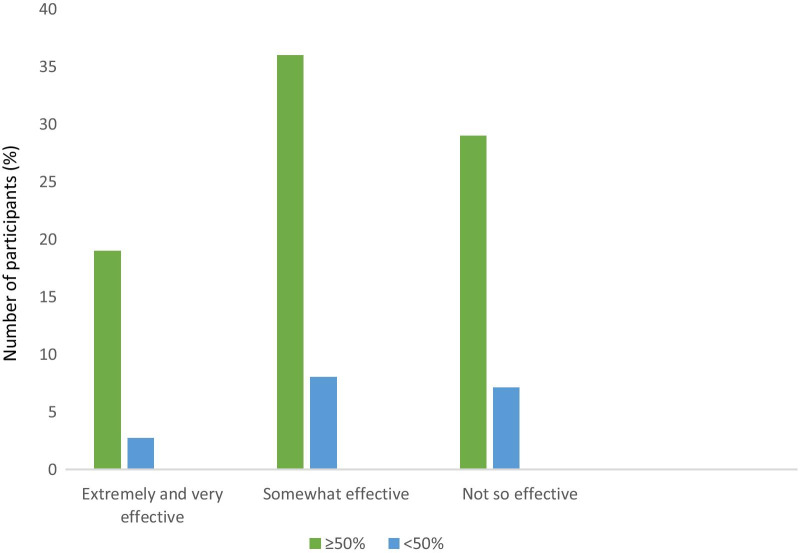
Bar graph demonstrating the responses to ‘how effective do you think crisis resonates with your clinical practice?’. This is sub-categorised by proportion of time spent seeing COPD patients (50% of their time including over and under

Upon ranking, the most preferred term was ‘flare-up’, followed by ‘lung attack’, ‘crisis’, ‘exacerbation’ and ‘chest infection’ (see Fig. 2). Respiratory nurses, who spent the majority (> 50%) of their clinical time in managing patients with COPD, infrequently preferred the term exacerbation. Upon direct questioning of preference between ‘exacerbations’ or ‘crisis’, the responses were equally split, with 49% respondents preferring ‘crisis’ compared to ‘exacerbation’. More than 60% of the nurses reported the word ‘crisis’ resonated with how a COPD acute event occurred in clinical practice (see Fig. 3) and the majority of these respondants were nurses who spent most of their time in clinical practice treating patients with COPD in different healthcare settings.

### Qualitative analysis

#### Understanding of terms

The survey responses clearly expressed that any term which is to be used by healthcare professionals needs to be easily undersood by patients and nurses. Existing terms such as ‘exacerbation’ and ‘flare-up’ were favoured namely because (1) they are already in use; (2) they are personal favourites; and (3) words already in current clinical practice offered the opportunity to educate patients, without the need to re-teach or re-learn a new term. Although ‘exacerbation’ was indicated to be a poorly understood term, a sub-theme was present for its advocacy. Respiratory nurses thought as the term ‘exacerbation’ is widespread, the challenge would be too difficult to change current practices. In exploring the other terms, ‘chest-infection’, ‘flare-up’ and ‘lung attack’, the common sub-themes within these included that; ‘chest-infection’ is not always relevant *(“for example when this is not treated with antibiotics”*); ‘lung attack’ is promoted by national charities such as the British Lung Foundation; while, ‘flare-up’ is commonly used by peers in other medical specialties (e.g. Rheumatoid Arthritis and Inflammatory Bowel Disease). Finally, although ‘crisis’ is a relatively new term, respiratory nurses asserted it was direct and implicit in encompassing the potential importance of the acute event that occurs in patients with COPD (see Table 1).

#### Psychological impact of terms

Respiratory nurses observed that the way a patient with COPD perceives a term was psychologically important. Currently used and proposed alternatives to the term ‘exacerbation’, namely ‘lung attack’, ‘flare-up’ and ‘crisis’ were considered to be emotionally charged. In particular, some respiratory nurses indicated that ‘lung attack’ appeared patronising to patients with COPD, while others stated ‘crisis’ was potentially an overreaction to the event.

#### Nursing perspective of how a patient will perceive new terms

The word ‘crisis’ received the most positive outlook in this theme. Furthermore, ‘crisis’ was associated with the most progressive perspective from respiratory nurses, with a view to using it in the future. General considerations included the historical meaning, familiarity in general use and other medical conditions (e.g. sickle cell crisis) and finally how it conveyed a sense of urgency. A further insight related to considering different terms for each method of healthcare utilisation and how there may be an associated expectation of terms (Table 2).

**Table 2:** Sub-themes and quotations of nursing perspective of how a patient will perceive new terms

Sub-themes	Quote
Meaning of ‘crisis’	‘*Crisis is a word of historical importance and has elements of time and cause attached to it’*.
	*‘Crisis is good for a bad exacerbation’*
Familiarity to the term ‘crisis’	*‘Haven't used the word crisis but actually may be a good way to recognise the importance’*.
Use of the term ‘crisis’ in other medical conditions	*‘I think that this fits with other diseases such as sickle cell’*
	*‘I think if we use the word attack or crisis it portrays the seriousness of the situation such as a heart attack as people understand this terminology’*.
Urgency of the word ‘crisis’	*‘It gives the idea that rapid intervention will be needed’*
	*‘A crisis or lung attack might be perceived as more concerning for a patient’*.
‘Exacerbation’ and healthcare utilisation	*‘Exacerbation not always infectious – using the word infection increases expectations for antibiotics’*

## Discussion

This is the first known attempt to understand the respiratory nurse perspective on use of the term ‘exacerbation’ albeit limited to the UK nursing perspective. In this survey, respiratory nurses reported that patients did not fully grasp the meaning of the term ‘exacerbation’. A minority of nurses indicated that the term ‘exacerbation’ was fully understood by patients, but it is widely known that a patient use of the term ‘exacerbation’ does not necessarily translate into comprehension [9, 10]. Concerns relating to health literacy, comprehension and usability of the term ‘exacerbation’ are echoed by patients [2] and healthcare professionals alike [9, 10]. Interestingly, our data showed a lack of unified consensus by respiratory specialist nurses on defining the term ‘exacerbation’, a problem that patients with COPD present with frequently. This is likely a consequence of the different guideline based concepts which define an acute ‘exacerbation’ of COPD [11, 12]*.* Although the majority of respiratory nurses defined ‘exacerbation’ in accordance with NICE guidelines which sees ‘exacerbations’ as symptom-based events, respiratory nurses also expressed other additional concepts related to treatment; which is likely affected by the GOLD guidance which has a more event-based definition [12]. Few nurses distinguished this difference, which is concerning if the lack of unified message may impact clinical care. For the nurses who reported ‘exacerbation’ was the preferred term, it was surprising to note their response was selected as a sense of surrender. Many respiratory nurses viewed any change in terminology was insurmountable which is in contrast to initiatives launched in other countries such as the collaborative effort national change in terminology from ‘exacerbation’ to ‘lung attack’ in the Netherlands [9, 10]. Nurses are uniquely placed in the patient journey to promote humanistic principles with patients who have chronic illness [13] and efforts to invigorate the resigned views held by the UK respiratory nurses surveyed will be important.

Our survey revealed an even greater discord between respiratory nurses’ opinions in the time-sensitive nature, patient perception and classification of the event itself. Respiratory nurses who focused on using symptoms for defining the acute event, were likely to place the onus of interpretation on a patient for the acuity of the event; and this was dependent on the respiratory nurse’s experience and preferences in communication. This may lead to a lack of clarity in patient management, which could have consequences in disease management for the patient [6]. Our survey cannot draw a conclusion as to whether this is a community or hospital nurse perspective preference due to the small sample size. However it has been recognised, that better outcomes are achieved in specialities where definitions of emergency situations are used [14]. Furthermore, it was apparent that on occasion, the nurse perspective was often paternalistic. This was especially regarding the terminology the nurse belived best represented the patients’ condition.

In reviewing commonly used alternative terms to ‘exacerbation’, we found the most favoured term in this survey was ‘flare-up’ and was a preferred term by nurses who report seeing patients greater than 50% percent of their time. ‘Flare-up’ was perceived to be less confrontational. This response is not surprising, as provision of emotional balance to a patient is traditionally considered to be an important nursing responsibility [13]. However this is in contrast to COPD patient preferences, with previous investigation showing that the term ‘flare-up’ trivialises the patients’ symptoms [15]. Furthermore, it was evident by the respondants that the term ‘flare-up’ indicated the biological inflammatory processes whilst simultaneously led to to gaps in interpretation, similar to the commonly used ‘chest infection’. Biologically, the terms ‘flare-up’ and ‘chest infection’ only explain a proportion of the underlying pathogenesis of COPD. In addition, respiratory patient aimed organisations use ‘flare-up’ in conjunction with adverbs such as ‘very’ to emphasise fatal acute events [14], with the implication that even patient aimed organisations recognise that the term ‘flare-up’ alone does not fully convery the urgency and seriousness of the acute event in patients with COPD. It can thus be concluded that the term ‘flare up’ has limitations to clinical settings, psychological impact and biological accuracy. Therefore, it can lead to confusion in expectations and frustration to both patients and nurses.

‘Lung attack’ was equally favoured in our survey. This term unlike ‘flare-up’, was associated with stronger emotional responses, where the term was commented to be forceful and exaggerated. It is likely that general reverence for the term ‘lung attack’, comes from its use in cardiology with heart attack [16]. However, a multicentre study on patient perspectives in COPD did not see lung attack to be the most preferred term [2]. In asthma, ‘lung attack’ is favoured by BTS/SIGN who promote its use [17] although opposition of ‘lung attack’ in COPD reflects the inherent differences in the population with COPD and disease processes [18].

The term COPD ‘crisis’ has only recently been championed as an alternative to ‘exacerbation’ [6], with a directive to healthcare professionals to pursue the underlying cause of the deterioration. In a previous patient survey, across 5 countries in Europe, ‘crisis’ was selected as the most favourable term by patients with COPD [2]. In our survey, respiratory nurses were more likely to reject a term which was unfamiliar to them. However, it was clear that when asked if ‘crisis’ resonated with clinical experience, patient understanding and literacy, the respiratory nurses were more likely to favour ‘crisis’ to ‘exacerbation’. Although some respiratory nurses expressed ‘crisis’ may be frightening to patients, it was considered equally beneficial to convey the seriousness of the situation. Some nurses commended the simplicity and directness of the word. Mastery of a simpler term, in varied clinical settings, is likely to enhance understanding across healthcare practitioners, patients and carers. Our respiratory nurses reported the term COPD ‘crisis’ immediately allowed nurses to consider the event as critical. This is especially important as outcomes following an acute deterioration of COPD are poor, particularly following a hospitalisation, where mortality is as high as 50% in 2 years [19].

## Strengths and Limitations

One limitation to discuss is that this survey was distributed in March 2020 and sampled 113 respiratory nurses with an unequal representation of nurses from each region in the UK and equally from different specialities. The lower response numbers was in part due to the ensuing COVID-19 pandemic in the UK, with the focus of the ARNS and PCRS organisations changing to the COVID-19 pandemic in the UK. Representation of survey enteries fell consequence to geographical COVID-19 surges [20] whilst the annoymised nature of the survey meant that the response rate is unknown. Despite this, this is the largest to date survey examining the UK respiratory nurses’ perspective on renaming COPD ‘exacerbations’ providing novel insights. A further limitation is our survey may be subject to recall bias as respiratory nurses were required to remember patient experiences with specific words over their clinical experiences. Nonetheless, we were reassured to see a significant amount of respiratory nursing experience was available to us. Finally, we sought to compare a fixed number of different terms for an ‘exacerbation’, which may inhibit identification of alternative newer terms. However, our choices were guided by preferences from patients with COPD [2] and we had to consider how long the survey will take. The validity of the survey was tested by “content validity” and the variables of interest and relevance were deemed appropriate by the authors, who have expertise in working with patients who have COPD. Furthermore, the qualitative data was regarded trustworthy as the channels which were used to distribute the surveys are reputable. In addition, the experiences of the authors were drawn to assess whether the responses were relevant to the topic at hand. A strength of this survey is that it was designed on the approach of “alternative-form reliability” by asking questions to test consistency in opinions from different perspectives. Generally, we found this revealed richer responses about the professional nurse opinions on the term ‘exacerbation’ in COPD.

## Conclusion

The impact which a respiratory nurse can have on a patient with COPD is significant, often repeated and occurs at times of heightened anxiety for a patient. Identification of the patients’ emotional needs can aid in building rapport and shared decision making. Currently, this is limited by the word ‘exacerbation’. The widely used term ‘exacerbation’, is defined and recommended by NICE guidelines [11] and international guidance [12]. There is thus limited exploration of the patient perspective from the nurse healthcare professional viewpoint which warrants exploration and has been attempted in our survey. Ultimately, these episodes of acute worsening of a patient with COPD have negative consequences in disease progression. Often this can lead to premature death [21, 22], posing significant health burdens in chronic illness [23]. Nurse-led initiatives in management of COPD indicate an optimistic outlook on reducing patient anxiety and unplanned doctor visits [24, 25]. However, these initiatives must also focus on effective patient communication, which is central to all patient encounters and a fundamental part of COPD management. Our survey highlights that the misinterpretation of the term ‘exacerbation’ between nurses is a cause for concern. In defining acute deterioration in COPD, alternative terms such as ‘crisis’ merit further exploration.

## Supplementary Information

**Additional file1.** Word cloud presenting psychological impact of ‘lung attack’ (**A**); ‘flare up’ (**B**); and ‘crisis’ (**C**). Majority of participants gave emotional responses. Generated from: https://www.jasondavies.com/wordcloud/

## Data Availability

The datasets used and/or analysed during the current study are available from the corresponding author on reasonable request.
