# Supplementary material for: Renaming COPD exacerbations: the UK respiratory nursing perspective
Source: BMC Pulm Med. 2021 Sep 23;21:299. doi: 10.1186/s12890-021-01662-9 (PMC8459136; doi:10.1186/s12890-021-01662-9)

**Supplementary table 1:**

Survey questions asked to all participants. In brackets is the options available to participants for responses.

| **Question number** | **Question** |
| --- | --- |
| 1 | What is your job role? (Free text) |
| 2 | What is your gender? (Free text) |
| 3 | What region do you work in? (Options were only available for the UK in multiple choice) |
| 4 | How many years of experience do you have as a respiratory nurse? (Free text) |
| 5 | How old are you? (Free text) |
| 6 | In your own words, please write the definition of an exacerbation in COPD, as you believe it to be? (Free text) |
| 7 | How often do you see COPD patients? (Likert scale) |
| 8 | When patients present to you with a deterioration in their COPD, do you ever use the word exacerbation with them? (Likert scale) |
| 9 | How often do your patients spontaneously use the word ‘exacerbation’ to describe their symptoms? (Likert scale) |
| 10 | Do you believe the word exacerbation adequately describes the way a patient feels when they have worsening symptoms of COPD? (Likert scale) |
| 11 | Do you think your patient understand the use of the word ‘exacerbation’? |
| 12 | If your patient does not understand the word exacerbation, which other words do you use to describe the event to patients instead? (Free text) |
| 13 | Of the following words-Lung attack, flareup, exacerbation, crisis and chest infection; which do you prefer to use? Rand in descending order (Ranking) |
| 14 | Please help us understand why you ranked the words as above. (Free text) |
| 15 | On a scale below, does the term COPD crisis adequately describe the worsening of symptoms of COPD to you? (Likert Scale) |
| 16 | On the scale below, how do you feel about using the term ‘COPD crisis’ compared to the word ‘COPD exacerbation’? (Likert scale) |
| 17 | On the scale below, how effective do you think the term COPD crisis resonates with your clinical practice? (Likert scale) |

**Supplementary figure 1:**

*Word cloud presenting psychological impact of ‘lung attack’ (A); ‘flare up’ (B); and ‘crisis’ (C). Majority of participants gave emotional responses. Generated from: https://www.jasondavies.com/wordcloud/*


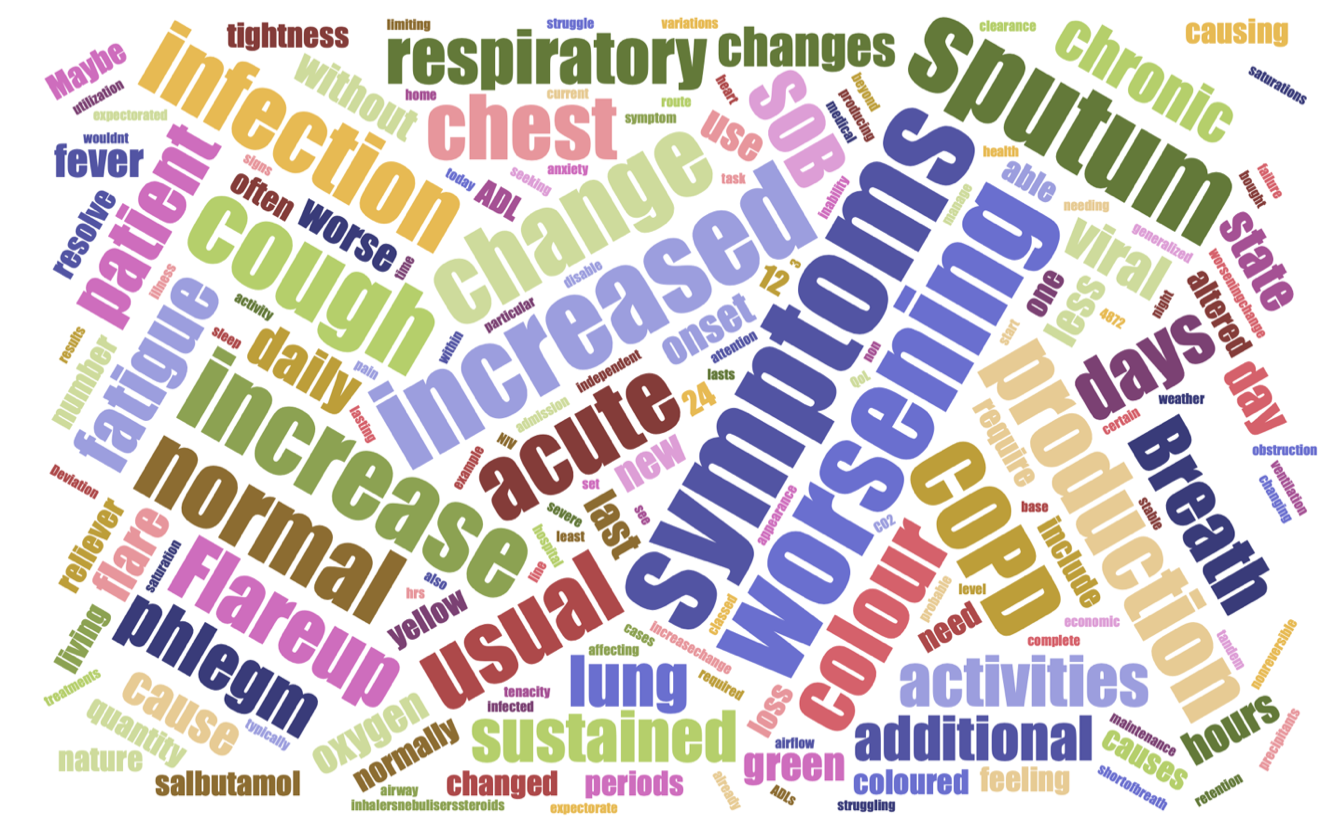

Supplement: Supplementary file 1 — Additional file1. Word cloud presenting psychological impact of ‘lung attack’ (A); ‘flare up’ (B); and ‘crisis’ (C). Majority of participants gave emotional responses. Generated from: https://www.jasondavies.com/wordcloud/ [file 12890_2021_1662_MOESM1_ESM.docx]
